# Systematic review protocol: The effects of theory-based interventions for self-help practices in the management of mild to moderate depression

**DOI:** 10.1371/journal.pone.0316960

**Published:** 2025-10-07

**Authors:** Asma’ Khalil, Zahir Izuan Azhar, Norley Shuib, Philip Baker, Xin Wee Chen

**Affiliations:** 1 Department of Public Health Medicine, Faculty of Medicine, Universiti Teknologi MARA, Sungai Buloh Campus, Selangor, Malaysia; 2 Ministry of Health, Malaysia; 3 Department of Psychiatry, Faculty of Medicine, Universiti Teknologi MARA, Sungai Buloh Campus, Selangor, Malaysia; 4 Faculty of Health, School of Public Health and Social Work, Queensland University of Technology, Australia; 5 Graduate School of Public Health, Yonsei University, Seoul, South Korea; University of Leeds Faculty of Medicine and Health, UNITED KINGDOM OF GREAT BRITAIN AND NORTHERN IRELAND

## Abstract

**Background:**

This protocol outlines a planned systematic review to assess the effects of theory-based interventions for self-help practices in managing mild to moderate depression. Depression is a leading global cause of disability and contributes significantly to the worldwide burden. Self-help practices present promising avenues for care. When grounded in psychological theory, these interventions may offer a structured and effective approach to symptom management. However, their effectiveness varies and often lack a theoretical foundation. The summarized evidence on theory-based interventions remains limited.

**Objectives:**

This review aims to evaluate the effects of theory-based self-help interventions in reducing the severity of depressive symptoms among adults with mild or moderate depression. It also aims to report any adverse effects associated with the use of these interventions.

**Methods:**

The review will follow PRISMA guidelines and is registered with the International Prospective Register of Systematic Reviews (PROSPERO), registration ID: CRD42024613188. Randomized controlled trials published between January 2014 and 2025 will be included in the analysis. Eligible theory-based interventions include Cognitive Behavioral Therapy (CBT), Acceptance and Commitment Therapy (ACT), and Mindfulness-Based Interventions (MBIs), delivered via digital platforms. Studies will be identified through searches of PubMed, Scopus, Web of Science, Science Direct, and Cochrane bibliographic databases.

Title and abstract screening will be completed within two months of the initial search, followed by one month for data extraction and two months for synthesis and analysis. Two reviewers will conduct screening, data extraction, and risk of bias assessment independently using the Cochrane Risk of Bias Tool (RoB 2). Key outcomes include changes in depressive symptom severity and adverse effects.

**Anticipated results:**

The planned synthesis will evaluate the effects of theory-based interventions, highlighting the anticipated contributions of theoretical frameworks to intervention outcomes.

**Expected impacts:**

The anticipated findings aim to inform future evidence-based guidelines for integration into primary care, supporting the development of accessible, scalable, and effective mental health solutions globally.

## 1. Introduction

Depression remains one of the leading global health concerns, contributing significantly to the overall disease burden. According to the World Health Organization (WHO), it affects over 280 million people globally, making it a top contributor to global disability in 2022 [1]. Primary care settings serve as the crucial first point of contact for individuals experiencing depressive symptoms, underscoring the importance of effective and accessible treatment options within this context [2].

Mild and moderate depression lie on the less severe end of the mental health continuum, differing from severe depression in both the symptom intensity and their functional impact. While mild to moderate cases may involve low mood, fatigue, and some social or occupational disruption, severe depression is characterized by pervasive symptoms such as suicidal ideation or psychosis, often requiring intensive clinical intervention [3,4]. Although pharmacological treatments are commonly used across the spectrum, they are associated with various challenges, including adherence issues and potential side effects [4].

Theory-based interventions in mental health are structured approaches grounded in psychological, behavioral, or social theories. These interventions target specific psychological mechanisms to influence attitudes, beliefs, and behaviors, promoting sustainable change in individuals [5]. Self-help practices refer to psychological or behavioral strategies individuals employ independently to improve their emotional and psychological well-being. These may include guided exercises, therapeutic writing, mindfulness activities, or structured modules [6].

Several theories are commonly used in advising self-help practices. For example, Cognitive Behavioral Therapy (CBT) is embedded in cognitive theory and focuses on identifying and restructuring maladaptive thought patterns and behaviors. CBT has demonstrated efficacy across various conditions, including anxiety, depression, and post-traumatic stress disorder (PTSD) [7]. Similarly, motivational interviewing, informed by self-determination theory, aims to enhance intrinsic motivation for behavior change, particularly in substance abuse and chronic health conditions [8]. Other examples include Mindfulness-Based Interventions (MBI) focus on fostering conscious, non-judgmental awareness of the present moment to enhance health and well-being [9], and family systems therapy, which emphasizes understanding and addressing complex relational dynamics within families, which influence individual behaviors and relationships [10].

The emergence of digital health innovations, including e-health and mHealth platforms, has revolutionized mental health care delivery. E-health refers to online platforms for healthcare services, while mHealth leverages mobile technology to provide interventions [11]. These platforms offer privacy, flexibility, and scalability, addressing barriers such as stigma and limited access to care, which are common challenges for individuals with depression [12]. Evidence supports the role of digital interventions in improving access, adherence, and outcomes for managing mild to moderate depression [13].

## 2. Rationale

Depression, particularly in its mild form, can significantly impact individuals’ quality of life. Theory-based non-pharmacological interventions, grounded in established psychological frameworks, could serve as effective modalities for managing mild to moderate depressive symptoms. Such interventions could provide valuable support to individuals awaiting specialist referral or as an alternative to pharmacological treatments, promoting accessibility and patient autonomy [14].

In recent years, digital self-help interventions for depression have proliferated, supported by increasing public demand, technological innovation, and global health system constraints [15]. Notably, despite the growing interest in digital self-help, there is limited evidence on the effectiveness of theory-based interventions in self-guided formats. Previous systematic reviews have generally evaluated self-help interventions without distinguishing whether they were grounded in psychological theory [16], and have often overlooked important variables such as theoretical foundations, delivery formats, or population-specific outcomes. Given this, it is essential to comprehensively evaluate the impact of self-help practices on reducing depressive symptoms. This includes exploring the methods, theoretical underpinnings, and intensity of interventions to understand their true effectiveness.

To address this knowledge gap, we propose a systematic review to evaluate the effects of theory-based interventions for self-help practices in managing mild to moderate depression. By synthesizing recent evidence from randomised trials, this review seeks to provide a comprehensive understanding of how theory-based interventions for self-help practices compare to pharmacological treatments and other therapeutic approaches in alleviating depressive symptoms and preventing relapse. This review will focus primarily on digital delivery modes, such as web platforms, mobile applications, and other e-health tools, due to their scalability, accessibility, and potential for autonomous use in theory-based self-help interventions without therapist-led facilitation.

### Research question (PICO framework)

Among adults aged 18 years and older with mild to moderate depression (P), what are the effects of theory-based self-help interventions delivered via digital platforms (I), compared to other interventions (C), in reducing depressive symptoms severity (O)?

## 3. Objectives

This systematic review aims to achieve the following objectives:

1. To evaluate the effects of theory-based interventions for self-help practices aimed at reducing the severity of depressive symptoms among adults aged 18 years and older with mild to moderate depression.
2. To identify and summarize any reported adverse effects associated with the use of these interventions.

## 4. Methods

### 4.1. Design and registration

This protocol has been registered with the International Prospective Register of Systematic Reviews (PROSPERO) (registration ID: CRD42024613188), and has the status of “registered” as of June 6, 2025. This systematic review will adhere to the Preferred Reporting Items for Systematic Reviews and Meta-Analyses (PRISMA) guidelines [17]. The review will concentrate on randomized trials, also referred to as randomized controlled trials (RCTs), that assess the effects of theory-based interventions for self-help practices in reducing depressive symptoms among adults with mild to moderate depression. The methodological approach includes a structured search strategy, defined eligibility criteria, data extraction, and risk of bias assessment procedures.

### 4.2. Information sources

A comprehensive search will be conducted across bibliographic databases, including PubMed, MEDLINE, Scopus, Web of Science, ScienceDirect, and Cochrane. The search will include peer-reviewed articles published from January 2014–2025, reflecting contemporary approaches. The term “contemporary approaches” refers specifically to the emergence and growing adoption of digital health technologies (e.g., mobile applications, online platforms) and the increasing integration of psychological theories (such as CBT, ACT, and MBIs) into self-help models during this period. Grey literature (e.g., dissertations, reports) and preprints will be excluded to ensure methodological consistency and data quality. Only studies published in English will be included, due to resource limitations for translation. This constraint will be acknowledged as a limitation.

### 4.3. Search strategy

The search will use keywords combined with Boolean operators (S1 and S2 Appendix). We will hand-search the reference lists of included studies for additional eligible studies. Authors will be contacted to obtain any missing or unpublished data, if applicable.

The search is structured in the following stages:

i Record Screening: The initial literature search has been completed, and title and abstract screening is currently ongoing. This stage is expected to be completed within two months of initiating the search.
ii Data Extraction: Once the full-text screening is complete, the data extraction process will begin. This process is expected to be finished within one month of completing the screening.
iii Anticipated Results Synthesis and Reporting: Data synthesis and analysis will occur immediately after the data extraction phase. The results of the systematic review are expected to be finalized and reported within two months following data extraction.

### 4.4. Eligibility criteria

The PICO framework will guide the study selection process:

#### • People (P).

- Adults aged 18 and older who are screened or diagnosed with mild to moderate depression and/or exhibit symptoms of depression.
- Specifically, a formal diagnosis of major depressive disorder based on the Diagnostic and Statistical Manual of Mental Disorders (DSM-5) or the International Classification of Diseases, 10th Revision (ICD-10), or
- Participants screened using commonly validated screening tools in the field of psychiatry, such as the Patient Health Questionnaire (PHQ-9), Depression, Anxiety and Stress Scale (DASS-21), Center for Epidemiologic Studies Depression Scale (CES-D), Beck Depression Inventory (BDI), or Hamilton Depression Rating Scale (HDRS). Cut-off scores defining “mild to moderate” will follow the original classification set by the developers of each screening tool. For example, scores between 5-14 for PHQ-9, scores between 16-26 for CES-D, and scores between 8-23 for HDRS [18–20].

These thresholds will be applied flexibly based on the instrument used and the operational definitions adopted by each included study.

- Participants must have experienced depressive symptoms for a minimum of two weeks, as defined by DSM-5 criteria or validated screening tools, to indicate a clinically relevant episode of mild to moderate depression [21].
- The participants may either be diagnosed with depression alone or with other medical comorbidities commonly associated with depression, such as chronic diseases.

#### • Intervention (I).

To be eligible, interventions must meet the following criteria:

- The intervention must be theory-based, which may include, but is not limited to, Mindfulness-Based Interventions (MBIs), Cognitive Behavioral Therapies (CBTs), Acceptance and Commitment Therapy (ACT), or Behavioral Activation Therapy (BAT) as primary interventions.
- The intervention must be delivered either as a standalone approach or as part of an integrated hybrid model, and it must exclude pharmacological treatment.
- The intervention must be administered through non-traditional formats, such as text, audio, video, CD/DVD, smartphone, computer, and the Internet (e.g., automated emails, web-based applications, automated phone calls, short text messages), or individual exercises like ‘therapeutic writing.’
- The intervention must be designed to be delivered independently, providing only limited support, guidance, or assistance from a therapist.
- The intervention must focus solely on self-help practices for individuals.
- The intervention may take place in clinical or non-clinical settings.

#### • Comparison (C).

- Pharmacological treatment, symptom monitoring, psychosocial intervention, therapist-led psychotherapy, and combination therapy will be included. In the synthesis, comparator groups will be classified based on the type of treatment (e.g., pharmacological, therapist-led intervention, or usual care).

#### • Outcomes (O).

The severity of depressive symptoms is evaluated using a validated assessment tool.

Studies involving any of these designs or intervention criteria will be excluded:

- RCTs without ethical approval from an accredited, recognized ethics committee or institutional review board.
- Non-randomised studies, observational studies, case studies, or commentaries.
- Studies that do not measure depression as an outcome.
- Studies that do not measure depression pre- and post-intervention.
- Long-term, open-label, uncontrolled follow-up of a randomized controlled trial (RCT).
- Interventions involving group-based support or regular contact with a therapist, coach, or other human support throughout the study period.
- Non-digital interventions or non-self-help formats.
- Studies that include pharmacological treatment as part of the intervention.

Studies involving any of these populations will be excluded:

- Participants diagnosed with depression and other psychotic or mood disorders, such as anxiety, mood disorder, bipolar disorder, or schizophrenia.
- Participants experiencing persistent, chronic, or treatment-resistant depression.

#### • Timing.

Outcomes will be measured at two time points to capture the effects of theory-based interventions for self-help practices in managing mild to moderate depression:

- Short-term outcomes: Measured within 0 to 3 months after the intervention.
- Medium-term outcomes: Measured 3 to 6 months post-intervention.
- Long-term outcomes: Measured from 6 months after the intervention onward.

This analysis will provide valuable insights into the durability and lasting impact of the intervention effects.

### 4.5. Study selection

The search results will be imported into EndNote, duplicate entries will be removed, and the remaining records will be imported into the Rayyan software for shared screening. The study selection process will follow a two-stage screening approach. In the first stage, two independent reviewers (AK, CXW) will review the titles and abstracts with the help of Rayyan’s machine learning capabilities. In the second stage, the full texts of potentially eligible studies will be evaluated against the predefined inclusion criteria. Any disagreements between the reviewers will be resolved by a third reviewer (ZIA). Reasons for exclusion at this stage will be documented.

A PRISMA flowchart will outline the study selection process, including the number of records identified, screened, excluded, and included in the review.

## 5. Data extraction and management

Data extraction will utilize a standardized form created in Microsoft Excel. Publications that describe the same study will be regarded as a single study. Two independent reviewers will extract relevant data from each included study. Any disagreements between the reviewers will be resolved through discussion, and if disagreement persists, a third reviewer will make the final decision. The data to be extracted includes:

- Study characteristics: author, publication year, country of conduct, and study design.
- Participant characteristics: sample size, age, gender, equity markers, and depression severity.
- Diagnostic criteria for depression (DSM-5 or ICD-10) and the screening tool used.
- Intervention details: type and intensity of interventions, duration, delivery method (in-person or online), resource usage, and process evaluations.
- Comparison group details: pharmacological treatment, symptom monitoring, psychosocial intervention, therapist-led psychotherapy, and combination treatment.
- Outcome measures and measurement tools: changes in depressive symptoms (e.g., PHQ-9, BD-II, or CES-D scale).

### 5.1. Study characteristics

A summary table will present the key characteristics of the included studies, including:

- Study design (e.g., RCT, randomized trial, single-arm intervention).
- Study settings: location and year of study.
- Sample size and participant demographics (e.g., place of residence, ethnicity, occupation, gender, religion, education, social capital, and socio-economic status).
- Study population/participants (e.g., adults with chronic diseases, HIV, cancer).
- Diagnostic criteria for depression (DSM-IV, ICD-10) and the screening tool used.
- Details of the intervention: theory-based approach, duration, delivery method, platform used, and resource usage.
- Comparator groups (e.g., pharmacological treatment, symptom monitoring).
- Outcome measurement tools (e.g., PHQ-9, BD-II, DASS-21, HAS, CES-D scale).

### 5.2. Outcomes: Effects of theory-based interventions

#### 5.2.1 Critical outcomes.

The primary outcomes of this systematic review will be the extent to which theory-based interventions influence the severity of depressive symptoms in adults with mild to moderate depression, as well as any adverse effects. The change in depressive symptom severity will be assessed using validated assessment tools commonly employed in mental health research. Examples of these tools include:

- PHQ-9: A self-reported measure that assesses the severity of depressive symptoms over the past two weeks.
- BDI-II: A widely used 21-item scale for evaluating depressive symptomatology.
- CES-D: A screening tool that measures depressive symptoms in general populations.
- HDRS: A clinician-administered assessment evaluating symptom severity.

Changes in depressive symptoms will be presented in terms of:

I Precision and statistical significance: Measured as the mean difference or percentage change in symptom scores from baseline to post-intervention, along with the effect size.
II Clinical significance: Defined as a pre-determined threshold of symptom reduction, such as a decrease of at least 50% on a scale or remission (e.g., achieving a PHQ-9 score <5) [22].

Adverse events reported in the trial will be identified and presented in frequency (percentage). This analysis will examine any reported adverse effects or unintended consequences, such as worsening symptoms, participant distress, or high dropout rates due to negative experiences. Evaluating the safety of interventions is essential to mitigate risks and build confidence in their use among healthcare providers and users [23].

#### 5.2.2 Important outcome.

To provide a comprehensive evaluation of self-help interventions for managing depressive symptoms, this systematic review will incorporate health equity considerations as an important outcome. This involves assessing the scalability and adaptability of the interventions across diverse settings, populations, and delivery platforms. Factors such as ease of integration into existing healthcare systems and the flexibility of the intervention to accommodate cultural, socio-economic, and demographic variations will be taken into account. These considerations are essential for understanding the potential for widespread use and the practicality of implementation in real-world scenarios.

### 5.3. Risk of bias assessment

The risk of bias in the included studies will be evaluated using the Cochrane Risk of Bias Tool (RoB 2) [24]. This is a comprehensive and standardized framework developed by the Cochrane Collaboration to assess the risk of bias in the results of RCTs, particularly concerning the design, conduct, and reporting of trials. The risk of bias assessment will be conducted independently by two reviewers (AK, CXW). Any disagreements between the reviewers regarding the risk of bias ratings will be resolved through discussion. If a consensus cannot be reached, a third independent reviewer (ZIA) will arbitrate the final decision.

The RoB 2 assesses five key domains that can introduce bias in RCTs:

i Bias arising from the randomization process.
ii Bias due to deviations from intended interventions.
iii Bias due to missing outcome data.
iv Bias in the measurement of the outcome.
v Bias in the selection of the reported result.

The risk of bias will be categorized into three levels. ‘Low Risk’ indicates that the trial is unlikely to be influenced by bias in the assessed domain. ‘Some Concerns’ suggests that there is a possibility of bias that could reduce confidence in the results. ‘High Risk’ implies that bias in the domain is likely to impact the reliability of the findings significantly.

### 5.4. Data synthesis

The systematic review will provide a comprehensive overview of the included studies. Data synthesis will include a meta-analysis if the included studies are sufficiently homogeneous in design, population, interventions, and outcome measures. However, variations in assessment tools, follow-up periods, and target populations may prevent meaningful statistical pooling. Therefore, a narrative synthesis will be performed to interpret the findings (aforementioned outcomes) and highlight patterns across the diverse evidence base. Findings will be summarized, with results presented in tabular format. The quality of evidence will be evaluated based on the GRADE framework, considering factors such as study design, risk of bias, precision, inconsistency, indirectness, and effect size. Evidence certainty will be categorized into four levels: “High,” “Moderate,” “Low,” and “Very Low” [25].

## 6. Discussion

Complex interventions for managing mild to moderate depression often involve multiple interacting components, contextual dependencies, and varied outcomes [4,26]. Theory-based interventions may integrate elements such as psychoeducation, cognitive restructuring, and behavioral activation, along with features to enhance user engagement [27]. This review may evaluate the individual and combined effects of these components and seek to understand user interactions with the digital platform and contextual influences like cultural norms and healthcare infrastructure that could influence interventions’ effectiveness, adaptability, and scalability [28,29]. This systematic review would perhaps address these dimensions of complexity, the component analysis, interaction assessment, and contextual evaluation, to provide a nuanced understanding of the factors influencing the success of theory-based interventions for depression.

### Health equity considerations

Incorporating health equity considerations is essential for evaluating digital interventions for mild to moderate depression. Health equity refers to eliminating avoidable disparities in health outcomes across population groups, influenced by factors like socioeconomic status, geographic location, ethnicity, and access to resources [30]. Digital interventions have the potential to overcome barriers such as geographic isolation and limited healthcare access, expanding their reach to underserved populations [31]. However, disparities in digital literacy, internet access, and the cultural relevance of intervention content may hinder the equitable distribution of benefits [32,33]. This review will assess the extent to which included studies address health equity by examining population characteristics (place of residence, ethnicity, occupation, gender, religion, education, social capital, and socio-economic position) [34]. Additionally, given the variability in individual responses, not all theoretical frameworks are equally effective for all users, necessitating careful adaptation and customization of interventions [35].

### Limitations and added value

Several limitations and potential challenges may arise from this planned review. A notable limitation of this review may be measurement bias from the common use of self-reporting of outcomes or self-declaration as having depression, without undergoing clinical assessment or receiving a confirmed diagnosis from a psychiatrist or other qualified mental health professional. This reliance may limit the certainty and comparability of findings [36]. Restricting the inclusion of English-language publications may introduce language bias and limit generalizability.

In addition, other important methodological challenges, such as potential heterogeneity in intervention approaches (e.g., types, delivery methods, and duration), theoretical frameworks (and their variability in theoretical fidelity), may impose a great challenge in synthesizing findings. Despite this limitation, the review is expected to offer significant contributions to the field of mental health interventions. It may provide a detailed analysis of interventions rooted in psychological theories, elucidating the foundational principles that underpin their design and effectiveness [37]. By exploring delivery methods such as web-based platforms and mobile applications, the review may offer detailed insights into how delivery formats influence engagement, adherence, and outcomes, catering to diverse user preferences [15,38].

### Expected impact

This planned systematic review is anticipated to provide a comprehensive evaluation of the role of self-help practices derived from theory-based interventions in managing adults with mild to moderate depression. Our review intends to highlight the novel findings where there may be certainty of theory-based interventions to reduce the severity of depressive symptoms. The anticipated results aim to inform future evidence-based guidelines for integration into primary care, supporting the development of accessible, scalable, and effective mental health solutions globally.

## Supporting information

S1 Appendix PRISMA-P (Preferred Reporting Items for Systematic review and Meta-Analysis Protocols) 2015 checklist: Recommended items to address in a systematic review protocol. (DOCX)

S2 Appendix Boolean operators search strategy. (DOCX)
