# Supplementary material for: Systematic review protocol: The effects of theory-based interventions for self-help practices in the management of mild to moderate depression
Source: PLoS One. 2025 Oct 7;20(10):e0316960. doi: 10.1371/journal.pone.0316960 (PMC12503273; doi:10.1371/journal.pone.0316960)
Supplement: S2 Appendix — (DOCX) [file pone.0316960.s002.docx]

**Supporting Information**

**S2 Appendix**

**Boolean Operators Search Strategy**

The search will be constructed using the same keywords as follows in all databases:

- depress* AND
- (theory OR theoret* OR model* OR principle* OR construct* OR framework*) AND
- (online OR web* OR digital OR mobile OR mHealth OR e-health OR educat OR intervention OR behavioural OR behavioral) AND
- (self-care OR self-help OR self-manage*)

Additionally, filter functions will be applied appropriately to each database, as suggested in the following table:

| **Database** | **Filter by** |
| --- | --- |
| Pubmed | - Publication date from 2014 to 2025 - Free full text - RCTs - English language - Human studies |
| Scopus | - Years 2014 to 2025 - Articles - English - All open access |
| Web of Science | - All open access - Publication years from 2014 to 2025 - Articles - English |
| Science Direct | depress AND (self-care OR self-help) AND (theory OR model OR principle) AND (online OR web OR digital)   - Publication years from 2014 to 2025 - Research articles - Open access and open archive |
| Cochrane | - Year first published from 2014 to 2025 - English - Cochrane trials |

**PubMed Search Strategy (Example)**

Database: PubMed (MEDLINE)

Date last searched: 16 September 2025

Search string: (((depress*[Title/Abstract]) AND (theory[Title/Abstract] OR theoret*[Title/Abstract] OR model*[Title/Abstract] OR principle*[Title/Abstract] OR construct*[Title/Abstract] OR framework*[Title/Abstract])) AND (online[Title/Abstract] OR web*[Title/Abstract] OR digital[Title/Abstract] OR mobile[Title/Abstract] OR mHealth[Title/Abstract] OR e-health[Title/Abstract] OR educat[Title/Abstract] OR intervention[Title/Abstract] OR behavioural[Title/Abstract] OR behavioral[Title/Abstract])) AND (self-care[Title/Abstract] OR self-help[Title/Abstract] OR self-manage*[Title/Abstract])

Filters applied: Free full text, Systematic Review, year 2014-2025
